# Artificial Intelligence for Diagnostic Guidance in Ocular Surface Disorders

**DOI:** 10.3390/jcm15051741

**Published:** 2026-02-25

**Authors:** Amr Almobayed, Omar Badla, Pragat J. Muthu, Diego Alba, Michael Antonietti, Anat Galor, Carol L. Karp

**Affiliations:** 1 Bascom Palmer Eye Institute, University of Miami Miller School of Medicine, Miami, FL 33136, USA; axa4141@miami.edu (A.A.); oxb248@miami.edu (O.B.); pjm189@med.miami.edu (P.J.M.); diegoalba@miami.edu (D.A.); mantonietti@mgb.org (M.A.); agalor@med.miami.edu (A.G.); 2 Department of Ophthalmology, Massachusetts Eye and Ear, Harvard Medical School, Boston, MA 02114, USA; 3 Department of Ophthalmology, Miami Veterans Administration Medical Center, Miami, FL 33125, USA

**Keywords:** artificial intelligence, deep learning, ocular surface disease, ocular surface tumor, dry eye disease, infectious keratitis, corneal ectasia, ocular surface squamous neoplasia, pigmented conjunctival lesions, pterygium

## Abstract

Artificial intelligence (AI) has been explored as a promising diagnostic aid for ocular surface diseases (OSDs). The spectrum of OSD ranges from highly prevalent benign conditions such as dry eye disease (DED) to rare but potentially dangerous disorders, including ocular surface squamous neoplasia (OSSN) and conjunctival melanoma. This review provides an overview of current applications of AI across the major categories of ocular surface pathology and specifically highlights anterior segment imaging modalities, including slit-lamp examination, optical coherence tomography (OCT), and in vivo confocal microscopy (IVCM). Meibography, tear film dynamics, biochemical profiling, and other DED-related measures are also examined. Across these domains, reported AI model performance matches or exceeds that of ophthalmologists, offering consistent, reproducible, and accurate approaches for guiding diagnosis. However, studies with limited external or prospective validation, variable labeling strategies, and small, device-specific datasets predominate in the current literature, thereby limiting generalizability. Large multicenter datasets, standardized diagnostic frameworks, multimodal integration, and prospective trials that assess human–AI cooperation in practical settings should be an emphasis in future research. By filling these gaps, AI systems could advance from experimental tools to clinically reliable applications that improve access and diagnostic accuracy in the care of ocular surface disease and tumors.

## 1. Introduction

The ocular surface includes the cornea, conjunctiva, and limbus, and its integrity is essential for maintaining vision and ocular comfort. It can be affected by a wide spectrum of disorders, with varying prevalence and dangers. Ocular surface disorders range from highly prevalent conditions, such as dry eye disease (DED) and infectious keratitis (IK), to less common but highly threatening conditions, such as ocular surface squamous neoplasia (OSSN) and conjunctival melanoma [1,2,3,4,5]. DED by itself is estimated to affect 5–50% of adults worldwide, depending on differences in diagnostic criteria [4]. IK accounts for an estimated 1.5 to 2.0 million new cases of monocular blindness each year, and corneal opacity remains among the leading global causes of vision impairment [6]. In contrast, OSSN and pigmented conjunctival neoplastic lesions are less common but can be far more dangerous due to their potential for invasion, recurrence, and systemic spread [7].

Accurate diagnosis of these diseases requires combining clinical examination with various imaging techniques. These traditional methods may show variability in sensitivity and specificity, particularly in early or subtle lesions. Interpretation can vary among observers, even among highly experienced clinicians and further influenced by inconsistencies in diagnostic criteria and guideline recommendations across regions. In addition, access to advanced imaging devices remains uneven worldwide [8].

**Figure 1:**
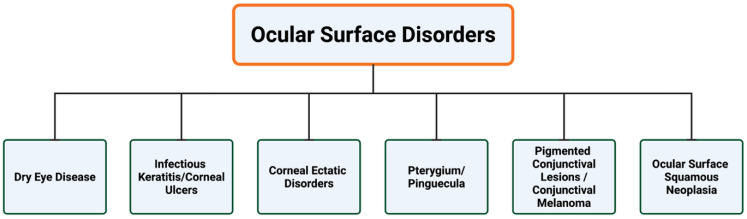
Schematic summarizing the ocular surface disease domains evaluated in this review. Artificial intelligence-based diagnostic applications are discussed across six major ocular surface disease categories, which form the organizational framework of the review.

These limitations may lead to underdiagnosis or delayed recognition, suggesting a need for diagnostic approaches that are more reproducible, objective, and scalable across settings. Artificial intelligence (AI), initially introduced into ophthalmology in 2016 through deep learning approaches applied to retinal disease screening [9,10], has been explored as a promising partner to help guide clinicians. In this review, we summarize the available evidence on AI applications to ocular surface disorders, covering both neoplastic and non-neoplastic conditions through a disease-based framework. The scope and structure of this review are summarized schematically in Figure 1**.**

## 2. Methods

A targeted search of the relevant literature was performed across PubMed and Google Scholar from 1 July 2017 to 30 September 2025. The search encompassed various combinations of “artificial intelligence,” “machine learning,” and “deep learning” with disease- and modality-specific keywords such as “ocular surface disease,” “dry eye disease,” “infectious keratitis,” “pterygium,” “ocular surface squamous neoplasia,” “conjunctival melanoma,” “anterior segment optical coherence tomography,” “in vivo confocal microscopy,” “meibography,” and related terms. Reference lists of key articles and prior reviews were reviewed to identify additional relevant studies not captured through database searches.

The inclusion criteria comprised original studies, systematic reviews, and meta-analyses published in English. Exclusions included self-standing abstracts and articles with duplicated data. Publications not meeting specific research criteria or lacking clear methods were also excluded. Data extraction involved three independent authors (O.B., P.J.M. and D.A.). Any inconsistencies were resolved through consensus discussions.

## 3. Dry Eye Disease

### 3.1. Clinical Background

DED is a multifactorial, heterogeneous ocular surface disease (OSD). The main characteristics of DED are tear-film instability, loss of homeostasis, and changes in osmolarity. It is sometimes associated with ocular surface inflammation and neurosensory abnormalities. In addition to the two broad classifications as aqueous-deficient and evaporative subtypes, DED subgroup classifications are being refined constantly [11]. Epidemiologic data from the United States, particularly the National Health and Wellness Survey, ref. [12] estimate that physician-diagnosed dry eye affects about 6.8% of adults. It is also worth acknowledging that this method may underestimate prevalence because it depends on self-reported symptoms with clinician confirmation and excludes individuals who do not seek care or interpret symptoms differently.

**Figure 2:**
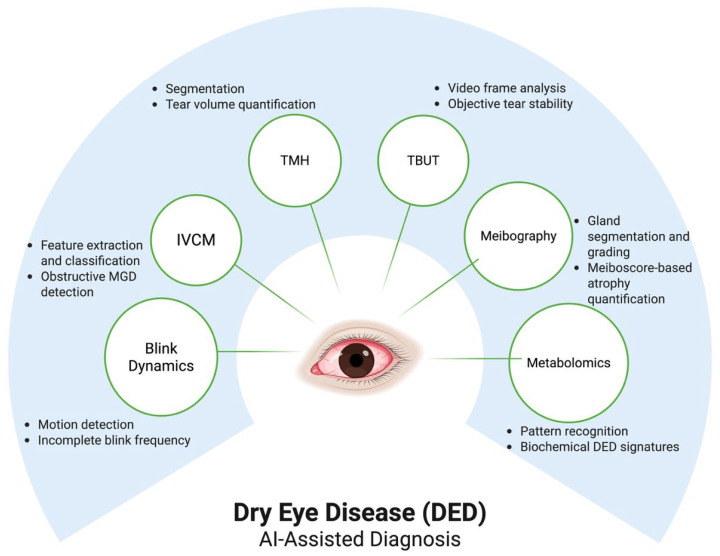
Artificial intelligence–assisted diagnostic domains in dry eye disease. Artificial intelligence (AI) approaches have been applied across multiple diagnostic tools in dry eye disease (DED), including tear meniscus height (TMH), tear film breakup time (TBUT), meibography, blink dynamics, in vivo confocal microscopy (IVCM), and metabolomic profiling. Across these domains, AI primarily supports automated segmentation, motion analysis, feature extraction, and pattern recognition to enhance the objectivity and reproducibility of dry eye assessment. This figure summarizes diagnostic domains and analytical tasks of AI-based approaches for DED discussed in this review.

Diagnosis and subtyping still rely on a combination of symptom-based questionnaires, such as the Ocular Surface Disease Index (OSDI), and clinical tests. These tests include tear breakup time, Schirmer measurements, ocular surface staining patterns, tear osmolarity, and imaging techniques such as meibography and tear meniscus height (TMH) [13]. These quantifiable features have provided the basis for the development of AI algorithms. Since 2017, deep learning-based approaches have been applied across nearly every aspect of DED diagnosis and sub-classification, each targeting specific components of the disease [14]. Across these diagnostic modalities, AI applications span multiple diagnostic modalities and focus on distinct elements of dry eye pathophysiology, including tear volume, tear film stability, glandular structure, eyelid dynamics, and biochemical profiling Figure 2.

### 3.2. Artificial Intelligence Applications in Dry Eye Disease Diagnosis

TMH is a well-established method to estimate tear volume. The downside of this method is that it is time-consuming and prone to variability due to manual caliper measurements. AI-based approaches appear to improve the objectivity and reproducibility of TMH assessment across imaging platforms, addressing these limitations. Stegmann et al. [15] trained a convolutional neural network (CNN) on 27,180 anterior segment optical coherence tomography (AS-OCT) (Optovue Inc., Fremont, CA, USA) images from 413 participants, with expert manual annotations as the reference standard. The model reached segmentation performance (Dice coefficient: 96.4%; pixel-wise accuracy > 99%) and demonstrated reproducible measurements across repeated scans. Nejat et al. [16] adopted a different approach, shifting TMH quantification to smartphone photography and training on 1021 images from 734 patients. They reported strong agreement with manual delineations from two cornea specialists (Dice coefficient: 98.68% and accuracy: 95.39%). More recently, Wang et al. [17] conducted a multicenter validation study using Keratograph 5M (Oculus, Wetzlar, Germany) imaging across five centers. The model achieved a mean intersection over union (MIoU) of 0.958 for color and 0.929 for infrared images. Correlation with clinician-annotated TMH measurement was strong, both internally (r = 0.935) and externally (r = 0.957). When reviewed together, these studies suggest that AI has the potential to quantify TMH with considerable accuracy across AS-OCT, smartphone, and Keratograph 5M imaging platforms.

Tear film breakup time (TBUT) remains one of the tests used to assess tear stability, though its interpretation can be variable [18]. Across multiple modalities, AI-based TBUT analysis has demonstrated diagnostic performance comparable to, and in some settings exceeding, expert clinician assessment by leveraging dynamic tear film features. Shimizu et al. [19] developed a CNN using more than 16,000 slit-lamp video frames captured with a portable smart eye camera. They compared the model’s predicted TBUT frames with clinician-annotated references and then assessed its diagnostic value for DED using the Asia Dry Eye Society (ADES) criteria (subjective symptoms + fluorescein TBUT ≤ 5 s recorded at slit-lamp). The model achieved 78.9% accuracy (Area under the curve (AUC): 0.877, sensitivity: 77.8%, and specificity: 85.7%), making it comparable to clinician grading.

Some investigators have moved beyond simple metrics and attempted to aid in DED diagnosis. Abdelmotaal et al. [20] applied deep transfer learning to ocular surface videos from the Keratograph 5M system. The goal was to distinguish patients with non-Sjögren’s aqueous-deficient dry eye, defined by OSDI ≥ 12, evidence of corneal or conjunctival staining, and abnormal tear stability or production. The best performing model achieved an AUC of 0.99 and ~91% video-level accuracy. Interestingly, this exceeded the performance of two cornea specialists, who achieved 86.5% and 71.0% accuracy, respectively. The results were comparable to those of a support vector machine trained on the full clinical dataset. One possible explanation for the superior AI performance is that the video-based approach captures subtle motion or surface cues that human readers tend to overlook.

Not all AI groups have relied on ocular surface videos. Another line of work has focused on lipid layer interferometry. Kikukawa et al. [21] trained a CNN on more than 9000 image patches from lipid layer interference videos captured by the KOWA DR-1α (Kowa Company, Ltd., Nagoya, Japan) interferometer. DED was defined by a Symptom Assessment in Dry Eye questionnaire score of >80 combined with a TBUT < 10 s. When evaluated against frame-level breakup annotations labeled by three engineers trained under the supervision of a dry eye specialist, the model produced an AUC of 0.898 (sensitivity: 84.3% and specificity: 83.3%). The accuracy increased to ~92% when the analysis focused on selected tear film regions.

Meibography is another widely used method for evaluating meibomian gland structure, particularly in evaporative DED, yet clinical grading of gland atrophy remains subjective and variable among observers [22]. This variability has motivated the development of AI-based approaches, which have demonstrated high agreement with expert annotations while reducing grading variability and analysis time. Wang et al. [23] developed a CNN to segment eyelid and atrophic regions from infrared meibography images. The dataset included 706 images from 467 adults, with 497 images for training and 209 for evaluation. From these segmentations, the model calculated percent atrophy and converted the values into standardized Meiboscores. Performance was compared with clinician-verified annotations that served as the reference standard. The model achieved 95.6% grading accuracy, outperforming the lead clinical investigator by 16% and the broader clinical team by 40.6%, while also substantially reducing analysis time. Segmentation accuracy was 97.6% for eyelids and 95.4% for atrophy regions; however, the model’s performance on gland segmentation was lower than for eyelid/tarsus boundaries, with an IoU of 66.7%. Collectively, these studies may suggest that AI can improve quantification of key DED-related metrics, such as meibomian gland atrophy and TBUT. It may help differentiate affected from unaffected eyes.

Other aspects of eyelid physiology may also contribute to DED and serve as targets for AI algorithm development. Incomplete blinking, for example, has been examined using automated methods [24]. AI models applied to blink dynamics have demonstrated high accuracy in detecting incomplete blinking and shown meaningful associations with established markers of dry eye severity. Zheng et al. [25] used a CNN to analyze blinking patterns from white-light videos recorded with the Keratograph 5M device. Their case–control cohort included 50 DED patients meeting tear film and ocular surface society dry eye workshop (TFOS DEWS II) criteria and 50 controls. Using 30 fps input, the model achieved 95.1% accuracy, with a sensitivity of 97.0% and a specificity of 93.2% against manual eyelid annotations. Lower-frame-rate videos at 8 fps were less sensitive to incomplete blinks and showed weaker relationships with clinical severity. Interestingly, incomplete blink frequency correlated with several severity markers, including NIBUT, tear osmolarity, conjunctival redness, and OSDI scores, thereby reinforcing the clinical value of capturing this behavior.

### 3.3. Emerging Approaches and Dry Eye Subtyping

Efforts to refine dry eye subtyping have continued to evolve. Tear Film Oriented Diagnosis (TFOD) attempts to move beyond the broad aqueous-deficient and evaporative labels, but its use in real clinics can be complex and often requires expert judgment [26]. Yokoi et al. [27] developed an AI-supported TFOD system that integrated videokeratography with a computed Blur Value to classify DED into four subtypes: severe aqueous-deficient (sADDE), mild or moderate aqueous-deficient (m/mADDE), decreased wettability (DWDE), and increased evaporation (IEDE). Fluorescein-based breakup pattern (FBUP)-based TFOD assignments served as the reference standard. The case–control dataset included 243 eyes. A 3D CNN extractor produced an overall accuracy of 78.4%. Accuracy by subtype was highest for sADDE at 92.3% for, followed by m/mADDE 79.3%, 75.8% for DWDE, and 72.7% for IEDE. These findings suggest that AI may support TFOD-based subtyping by standardizing classification in an otherwise expert-dependent framework.

In vivo confocal microscopy (IVCM) (HRT III with Rostock Cornea Module, Heidelberg Engineering GmbH, Heidelberg, Germany) offers microscopic detail of the eyelid and ocular surface, enabling AI-based detection of meibomian gland abnormalities that are difficult to assess with routine clinical methods. Using IVCM images, AI models have demonstrated high diagnostic accuracy for identifying obstructive meibomian gland dysfunction (MGD), supporting its role as a quantitative adjunct to expert interpretation [28,29]. Maruoka et al. [30] trained several CNNs, including DenseNet-201, DenseNet-169, VGG16, and InceptionV3, on confocal images from 221 individuals, 137 with obstructive MGD and 84 with normal glands. Labels were based on the consensus of three eyelid specialists. DenseNet-201 performed the best, achieving an AUC of 0.966 with a sensitivity of 94.2% and specificity of 82.1%, compared with consensus clinical diagnoses. An ensemble approach further improved performance, reaching an AUC of 0.981, with a sensitivity of 92.1% and a specificity of 98.8%. These findings demonstrate that AI has the chance to reliably detect glandular abnormalities on IVCM images.

Beyond imaging, investigators have explored biochemical profiling for DED. Early evidence suggests that metabolomic signatures can differentiate DED from healthy states, although performance remains modest and data are limited. Amouei-Sheshkal et al. [31] examined targeted metabolomic data from cataract surgery with and without dry eye, defined by TFOS DEWS II criteria. They evaluated 8 deep learning models using aqueous humor metabolite profiles from 54 patients with DED and 27 controls. Logistic regression performed best, with an AUC of 0.838 and balanced accuracy of 0.735, exceeding the performance of baseline dummy classifiers and the other tested models. The results may suggest that metabolic signatures could complement imaging-based assessments.

### 3.4. Summary of Dry Eye Diagnostics

AI can bring a degree of objectivity that is often hard to achieve with traditional dry eye assessments. Technology has been applied across a wide range of domains, including TMH and TBUT analyses, meibography, blink dynamics, metabolomic profiling, and AI-assisted subtyping. The future holds promise for AI-related DED diagnostic tools.

## 4. Infectious Keratitis/Corneal Ulcers

IK is an important cause of corneal opacification and preventable blindness worldwide. Multiple microorganisms can cause IK, including bacteria, fungi, viruses, and parasites, and their prevalence varies across geographic regions and risk factor profiles [5]. The diagnosis of IK relies on having an informative clinical history and examination supplemented by corneal scraping, microscopy, staining, and culture. However, cultures require several days to return results, which may limit their utility when clinicians need to make immediate treatment decisions [32]. These practical challenges have prompted several groups to develop AI-based diagnostic approaches that might accelerate recognition and improve accuracy [33]. Across multiple studies, AI models have consistently demonstrated the ability to detect IK from slit-lamp photography with diagnostic performance comparable to, and in some cases exceeding, that of experienced ophthalmologists.

Gu et al. [34] trained a CNN using 5325 slit-lamp photographs of clinically diagnosed IK. Diagnostic labels were assigned by consensus of cornea specialists at tertiary centers and prospectively compared against that of ten ophthalmologists. Sensitivity and specificity were equal to, and in some comparisons exceeded, clinician performance, with an AUC of 0.939 for IK. That said, steps need to be taken to address misclassification of corneal dystrophies or degeneration as IK.

Beyond binary detection, AI systems have also shown utility in clinically relevant differentiation tasks. Tiwari et al. [35] took a different angle by fine-tuning a VGG-16 network using images from the Steroids for Corneal Ulcers (SCUT) and Mycotic Ulcer Treatment (MUTT) trials. Their goal was to distinguish active ulcers from healed scars. The dataset included 612 patients for training, 200 for validation, and 200 for testing. On the main test set, the model achieved 93.5% sensitivity, 84.4% specificity, a 92.0% F1 score, and a 0.973 AUC. External validation at Stanford yielded an AUC of 0.947. Performance was comparable to that of cornea specialists but was limited to bacterial and fungal infections.

A broader, multicenter approach was pursued by Redd et al. [36] from four centers in India. They analyzed 980 microbiologically proven ulcers, including 500 fungal and 480 bacterial. A MobileNetV2 model achieved AUCs of 0.86 (single-center external) and 0.83 (multicenter), outperforming an ensemble of 12 cornea specialists (AUC 0.76). Accuracy was higher for fungal (81%) than bacterial (75%) ulcers.

Building on this same multicenter dataset, Soleimani et al. [37] developed three tailored models. The first one was for primary care physicians to distinguish ambiguous IK, and the second two were for experts to classify fungal keratitis and its subtypes. To reduce label leakage, training was restricted to a single site, whereas evaluation was performed on both a single-center external and a multicenter test set. Under this framework, MobileNetV2 achieved performance metrics comparable to those reported by Redd et al., including AUCs of 0.86 and 0.83, again exceeding the performance of the cornea specialists.

Beyond slit-lamp photography, AI has also been applied to advanced imaging. In IVCM models, Essalat et al. [38] trained on 4001 microbiologically confirmed images obtained by smear and culture, achieving ≥90% accuracy, precision, recall, sensitivity, specificity, and F1 scores for fungal and acanthamoebic keratitis. While AS-OCT has been explored for 2D and 3D reconstruction of IK lesions, its applications remain in early stages. AI-based diagnostic approaches for IK use are summarized in Table 1.

Taken together, these studies demonstrate the potential of AI to expedite the diagnosis of IK through triage and telemedicine, particularly in remote regions or settings with limited access to specialists [36]. Despite these promises, current challenges associated with IK models remain, which need to be addressed before they can be successfully deployed in a clinical setting on a broader scope [39,40,41].

## 5. Corneal Ectatic Disorders

Early detection of corneal ectatic disorders, such as keratoconus (KC) and post-refractive surgery ectasia, is critical in preoperative screening for refractive surgery [42]. Traditional diagnostic strategies still rely on keratometry, pachymetry, and tomographic analyses [43]. Each one of these tools captures only one part of a complex process. This limitation may explain the growing interest in AI as a means of integrating multiple parameters and synergistically improving sensitivity for detecting early or subclinical diseases [44].

To build on this idea, Ambrosio Jr et al. [45] combined Scheimpflug tomography using a Pentacam HR (Oculus Optikgeräte GmbH, Wetzlar, Germany) with corneal biomechanical parameters to generate the Tomographic and Biomechanical Index (TBI). Their dataset included 850 eyes and comprised 480 normal eyes, 204 keratoconic eyes, 72 eyes with no surgery and very asymmetric ectasia, and 94 healthy contralateral eyes of patients with very asymmetric ectasia. Ground truth was confirmed by corneal specialists through clinical examination and tomographic imaging. Using this framework, the model achieved an AUC of 0.996 for identifying both clinical and subclinical ectasia, a result that appears to underscore the value of combining structural and biomechanical data.

In subsequent work, Lopes et al. [46] introduced the Pentacam Random Forest Index (PRFI), which was trained on a larger cohort that included 2980 stable laser in situ keratomileusis (LASIK) eyes, 45 post-LASIK ectatic eyes, and 182 keratoconic eyes. The PRFI was validated using two independent datasets: 298 stable LASIK eyes and 188 very asymmetric ectatic eyes. The PRFI achieved an AUC of 0.992, comparable to that of TBI. Notably, this model appeared particularly effective in identifying susceptibility to post-LASIK ectasia, whereas the TBI was more sensitive to very early or subclinical disease [45,46]. This difference likely reflects the way each index weighs tomographic versus biomechanical inputs.

Building on the initial TBI framework, Ambrosio Jr et al. [47] later published a second phase of their study that expanded both the dataset and the disease spectrum. The updated model was trained using 1680 normal eyes, 1181 keratoconic eyes, 551 healthy contralateral eyes of patients with very asymmetric ectasia, and 474 unoperated ectatic eyes. As in earlier work, ground truth labeling relied on clinical and tomographic confirmation. The model achieved an AUC of 0.999 for clinical ectasia and 0.945 for subclinical disease, exceeding the performance of the original version. A related study by Huo et al. [48] adopted a different approach, developing ethnicity-specific models, highlighting that diagnostic performance may depend on population-level differences that are often overlooked in AI training datasets.

Beyond detection, there has also been interest in creating an AI model for disease staging. Herber et al. [49] proposed a random forest model to stage KC using dynamic corneal response parameters and pachymetric data. The Topographic Keratoconus Classification (TKC) derived from Pentacam indices served as the reference standard. Their model achieved AUCs of 0.97 for healthy eyes, 0.88 for mild, 0.89 for moderate, and 0.95 for advanced keratoconic eyes. These results suggest that AI-based staging may support more objective monitoring of disease severity and progression over time. Key AI-based approaches for the detection and staging of corneal ectatic disorders are summarized in Table 2.

Although these different models illustrate the potential of AI to strengthen both early detection and staging of corneal ectatic disorders, their translation into routine clinical practice remains limited. For example, ethnic diversity within the training models is often underrepresented. Addressing this limitation, along with others, will be necessary before such algorithms can be confidently incorporated into refractive surgery screening and long-term KC management [50].

## 6. Pterygium/Pinguecula

Pterygium is a fibrovascular proliferation that extends from the conjunctiva onto the cornea, whereas pinguecula represents a yellowish degenerative change that remains anatomically confined to the conjunctiva [51,52]. Both are benign lesions and are strongly associated with chronic ultraviolet exposure [53,54,55,56,57]. On AS-OCT, pterygium appears as a hyperreflective subepithelial lesion advancing onto the cornea, while pinguecula is usually seen as a more localized, well-defined conjunctival lesion that terminates at the limbus [58,59,60,61]. In routine practice, these entities are generally diagnosed clinically. In select cases, imaging and histopathologic evaluation are important, particularly when the appearance is atypical or there is concern for malignancy. These lesions may masquerade clinically as other diseases, as pterygium coexists with OSSN in up to 14.96% of excised specimens, underscoring the limits of clinical examination alone [62,63,64,65,66].

AI applications in this area have followed a logical progression. Early studies relied on standard clinical photographs, whereas more recent efforts have begun to incorporate higher-resolution modalities, such as AS-OCT and histopathology (Table 3). Together, these approaches provide a framework for not only diagnosis but also grading and characterization across diagnostic levels. Overall, existing studies consistently show that AI models can accurately detect and grade pterygium from clinical photographs, with performance that approaches expert evaluation, while applications to advanced imaging and histopathology remain comparatively limited. Several groups have used slit lamp and smartphone images to detect pterygium and stratify severity based on lesion size and corneal involvement. Hung et al. [67] proposed a multistage deep learning system trained on 1054 anterior-segment photographs, annotated by two corneal specialists with consensus review. Their workflow mirrored clinical reasoning: first identifying the presence of pterygium, then localizing the margins and cuts, and finally staging early disease versus advanced disease. Performance was high across all stages, which may suggest that the aforementioned workflow helped the model handle variability in lesion appearance.

Wan et al. [68] addressed the problem using a larger, more heterogeneous photographic dataset. Their model was trained on 2855 slit-lamp images spanning four diagnostic categories: 1312 healthy eyes, 251 subconjunctival hemorrhage, 909 observation-stage pterygium, and 383 surgical pterygium. The model achieved 97.9% overall accuracy and an AUC of 0.98, with a sensitivity of 92.1% and a specificity of 99.0% for surgical-stage pterygium. By contrast, few AI studies have focused on AS-OCT or IVCM, even though these modalities are commonly used by clinicians to evaluate pterygium [59,61,66]. Greenfield et al. [69] demonstrated excellent binary classification of OSSN versus pterygium or pinguecula using AS-OCT images. The limited number of OCT-based AI studies likely reflects the additional complexity of acquiring and annotating these datasets rather than a lack of clinical relevance.

Beyond surface imaging, AI has also been applied to histopathologic assessment. Kim et al. [70] reported the first automated grading of pterygium histopathology using AI. Their dataset comprised 400 microscopy images from 40 specimens, each graded on a standard histopathologic scale from I to IV. A decision tree classifier achieved accuracy between 75–82% on an independent test set and demonstrated an 81% true positive rate for multi-grade classification on external validation. While clearly a proof of concept, this work suggests that AI can capture subtle histologic features such as inflammatory changes, vascular density, and elastotic degeneration, which may eventually inform recurrence risk. Comparable AI studies for pinguecula are notably absent, likely because excision is uncommon unless lesions appear atypical or are concerning for malignancy.

A recent systematic review and meta-analysis by Tiong et al. [71] provides a broader view of the field. They analyzed 20 studies encompassing 45,913 images on deep learning applications for pterygium. For automated detection, pooled results from 14 studies involving 8094 images showed a sensitivity of 98.1% and a specificity of 99.1%. Severity grading, evaluated across eight studies with 1631 images, yielded a pooled sensitivity of 91.2% and a pooled specificity of 92.9%. In most cases, expert ophthalmologist grading served as the reference standard, although only one study directly compared AI performance with that of clinicians. While these results appear impressive, most models were tested only on internal datasets. 19 of the 20 studies lacked external validation, and many relied on case–control designs using clearly normal and overtly diseased images. This approach may inflate reported performance by excluding borderline presentations that clinicians routinely encounter.

Overall, AI has demonstrated strong performance in the detection and grading of pterygium, particularly when applied to slit-lamp and photographic datasets. Early work has evaluated the use of AI to analyze AS-OCTs. Evidence remains limited regarding pinguecula or confocal imaging. Future studies will hopefully better evaluate disease progression and aid in differentiating it from malignant masquerades.

**Table 3:** Summary of studies using AI for pterygium detection and grading.

Reference Number	Study (Year)	AI Task	Dataset	Key Performance	Summary
[72]	Mesquita et al. (2012)	To monitor the progress of pterygium (pre-AI algorithm)	58 slit lamp images of eyes with pterygium (Brazil)	Correct pterygium segmentation rate 63.4%.	Without a deep learning, they demonstrate feasibility of image-based pterygium progression measurement; performance affected by lighting and subtle disease advancement.
[73]	Wan Zaki et al. (2018)	To classify pterygium vs. normal	3017 anterior segment images (multi-country)	AUC 0.956.	Demonstrates feasibility of computer-aided pterygium screening using handcrafted features; no automated grading system implemented.
[74]	Zhang et al. (2018)	Classify multiple OSDs, including pterygium, interpretable multi-class AI system	1513 images (China)	Overall accuracy 93%; pterygium classification accuracy >95%.	Explainable multi-disease AI system with strong performance; lacks multilabel classification and fine-grained semantic segmentation.
[75]	Saad et al. (2019)	To classify pterygium vs. normal	844 images (Malaysia)	Accuracy 94.1%; AUC up to 0.95.	Has a good internal performance on CNN-based pterygium detection.
[76]	Zulkifley et al. (2019)	To classify pterygium vs. normal, plus pterygium localization	120 original images, augmentation to 3000 images (Australia)	AUC 0.970.	Improves pterygium detection and localization over traditional methods using deep learning; relies on limited training data.
[77]	Zamani et al. (2020)	To classify pterygium vs. normal (multiple CNN models)	386 images (Malaysia/Brazil)	Best model in the study had an AUC of 1.00	High performance CNN-based pterygium detection using transfer learning; results derived from internal cross-validation only.
[78]	Abdani et al. (2021)	To classify pterygium vs. normal	328 images (Australia)	AUC 0.987; accuracy 93.3%.	Accurate deep learning segmentation of pterygium across disease stages.
[79]	Xu et al. (2021)	To classify pterygium vs. normal and grade severity (2-class)	1220 images (China)	Detection: Sens 99.6%, Spec 100%. Grading: Sens 92.7%, Spec 90.6%.	Demonstrates strong agreement in pterygium detection and severity grading to expert diagnosis.
[80]	Zheng et al. (2021)	To classify pterygium vs. normal and grade severity (“observation” (mild) vs. “surgery” (severe) required)	4984 images (China, Nanjing Eye Hosp.)	Detection AUC 0.976; grading AUC 0.872 to 0.891.	Enables pterygium screening and grading for resource-limited clinical settings.
[81]	Fang et al. (2022)	To classify pterygium vs. normal and grade severity	3132 images (2106 pts)—Singapore; Grade severity—(2 external test sets)	Internal test: AUC 0.995. External Test1: AUC 0.991. External Test2: AUC 0.997.	Demonstrates high accuracy in both detection and grading. Had two external validations. Performance dropped on one external set (spec 87%) likely due to data differences.
[82]	Gan et al. (2022)	Identify pterygium requiring surgery (binary: surgery vs. observation)	172 anterior-segment photos (China)	Accuracy 94.12%, AUC 0.980.	Predicts surgically referable pterygium from anterior segment images with interpretable activation maps. Suggests an effective tool for referring surgical cases.
[67]	Hung et al. (2022)	To classify pterygium vs. normal and grade pterygium severity (3-class)	237 images (134 pts, Taiwan)	Detection accuracy 91.7%; grading (3-class) mean accuracy 88.6%.	Uses multistage deep learning system for pterygium detection and grading. Chose frontal and lateral view slit lamp photographs to potentially be widely applied.
[83]	Zhu et al. (2022)	To classify pterygium vs. normal	1034 images (China)	AUC 0.99.	Uses VGG16 CNN; excellent performance on internal split; no external validation.
[84]	Zamani et al. (2023)	To classify pterygium vs. normal	1080 images (Malaysia)	Best model AUC 0.996 (10-fold cross-validation)	Improves pterygium detection under limited data conditions using patch-based deep learning classification.
[70]	Kim et al. (2023)	To grade pterygium (I to IV)	400 immunohistochemistry images from 40 patients, 10 sections per specimen, two hospitals	Internal AUC 0.918; external AUC 0.87 for histopathologic grading.	First AI-enabled quantitative histopathologic grading of pterygium demonstrates feasibility. Although it is multicenter, it’s limited by its small sample size.
[85]	Kumar et al. (2023)	Grade pterygium severity (multi-class)	150 images (public + India)	Accuracy 96.6% on test set.	Compared ML methods; CNN-based grading outperformed Back Propagation Neural Networks (BPNN) for pterygium severity assessment; conference-level reporting.
[68]	Wan et al. (2023)	To classify normal, subconjunctival hemorrhage and pterygium (observe vs. surgery) 4-class total	2855 images (China); 4-class: normal, subconjunctival hemorrhage, pterygium (observe vs. surgery)	Detection AUC 0.98; surgical-stage sensitivity 92.1%, specificity 99.0%.	Distinguishes surgical versus observational pterygium with a high-performing multi-class model, supporting screening in resource-limited settings.
[86]	Liu et al. (2024)	To classify pterygium vs. normal and grade pterygium severity (2-class)	22,081 images (China, smartphone)	Detection: AUC ~1.00. Grading: AUC 0.936–0.968. Expert vs. AI: Ophthalmologists 98.5% vs. model 98.5% (diagnosis); 93.9% vs. 88.5% (grading).	Fusion model on smartphone images (ResNet101 + segmentation) achieved expert-level accuracy. Note: No external set (all internal); only study directly comparing to clinicians (showed comparable performance).
[87]	Luo et al. (2024)	Classify multiple OSDs, including pterygium vs. normal	953 images (China)	Pterygium detection AUC 0.96.	Enables OSD screening using smartphone-based model, though performance is limited by image quality and class imbalance.
[88]	Ticlavilca-Inche et al. (2024)	To classify pterygium vs. normal and grade pterygium severity (2-class)	534 images (Peru)	Detection AUC 0.96; grading sensitivity 74.4%, specificity 100%.	Achieves high diagnostic accuracy for pterygium using transfer learning, but grading sensitivity remains limited.
[89]	Wu et al. (2024)	To classify pterygium vs. normal and grade pterygium severity (2-class)	4595 images (China)	Detection: Accuracy 99.79%, AUC 1.000. Grading: Accuracy 92.8%.	Combines MobileNetV2 with a self-attention mechanism to achieve high-performance pterygium detection and severity grading outperformed earlier models (Hung 2022) [67]. Has excellent accuracy and efficiency suitable for low-resource settings.
[90]	Moreno-Lozano et al. (2024)	To classify pterygium vs. normal	1000 images (Peru/Ecuador)	Best model (Se-ResNeXt50): Accuracy 92%.	Benchmarking study showing that SE-ResNeXt50 outperformed several modern CNN architectures for pterygium detection. Findings support potential mobile deployment.
[91]	Ji et al. (2025)	Automatic pterygium segmentation & grading (continuous measurement of invasion)	(Harbin, China; dataset size not stated)	Grading (severity stage): Accuracy 93.60%, with strong agreement with specialists (κ ≈ 0.89)	Proposes a two-stage system integrating deep learning segmentation with quantitative curve-fitting to enable standardized pterygium grading and invasion measurement. Demonstrates strong agreement with specialists and high grading accuracy, but is limited by manual annotations, and restricted imaging conditions.
[92]	Li et al. (2025)	Multimodal AI (large language model (LLM) + image)—detect 3 OSDs: grade keratitis & pterygium	375 images (290 eyes, smartphone)	Few-shot Learning Results: Pterygium grading accuracy 66.7% (with 5-shot); improved with more examples (performance increased significantly, *p* < 0.01). Detection of any OSD: 86.96% (zero-shot).	Comines vision models and LLMs for OSD detection and grading using smartphone images. Highlights potential of LLM-based tools; requires further training for higher accuracy.
[71]	Tiong et al. (2025)	Summary of DL accuracy for pterygium (14 studies in diagnostic meta-analysis; 8 in grading meta-analysis)	20 studies, 45,913 images total	Detection: pooled sensitivity 98.1%, specificity 99.1%. Grading: sensitivity 91.2%, specificity 92.9%.	Meta-analysis showing pooled high diagnostic accuracy of deep learning models for pterygium detection and grading. Most included studies lacked true external validation and population diversity.

While the current AI studies for pterygium demonstrate high accuracy, their real-world utility remains limited. One specific limitation is that lesion size definitions vary substantially across datasets. Standardizing these definitions is necessary before these tools can be translated into routine clinical practice.

## 7. Pigmented Conjunctival Lesions/Conjunctival Melanoma

Pigmented conjunctival lesions span a wide spectrum of diagnoses, ranging from benign lesions such as nevi to malignant lesions, for example, conjunctival melanoma. Nevi are the most common conjunctival melanocytic lesions. They present at a younger age and have a low malignant potential. In comparison, primary acquired melanosis (PAM) with atypia has an increased risk of progression to melanoma. Conjunctival melanoma is rare, with an estimated annual incidence of 0.2 to 0.8 cases per million. However, its clinical behavior is often aggressive [3].

Currently, diagnosis still relies primarily on slit lamp biomicroscopy. Adjunctive imaging, such as AS-OCT or IVCM, can aid in evaluating epithelial or subepithelial involvement and in visualizing atypical melanocytes, respectively. Histopathology remains the gold standard when malignancy is suspected [93].

While AI applications in this area remain limited, one recent study directly examined melanoma detection. Yoo et al. [94] developed a deep learning model using slit-lamp photographs, employing generative adversarial network (GAN)-based augmentation and transfer learning to classify pigmented conjunctival lesions. Their dataset comprised 398 slit-lamp images, including 136 images of melanoma, 93 images of nevus/melanosis, 75 images of pterygium, and 94 images of normal conjunctiva. They used an additional 100 smartphone-captured 3D phantom melanoma images for external validation. Ground truth labels were determined by ophthalmologists through clinical or histopathologic confirmation. Using a MobileNetV2 architecture, the model achieved an AUC of 0.983 with an accuracy of 97.2% for binary melanoma detection, 87.5% for four-class classification, and 94.0% on external phantom validation. These results suggest that AI-assisted screening for conjunctival melanoma using consumer-grade imaging devices may be feasible, at least in controlled settings.

There is a lack of primary AI models for conjunctival melanoma, potentially due to its rarity, with only one published study to date using camera-based images. Future studies will hopefully evaluate the role of OCT, IVCM, or histopathology images [95].

## 8. Ocular Surface Squamous Neoplasia

OSSN refers to a spectrum of lesions ranging from dysplasia and intraepithelial neoplasia (carcinoma in situ) to invasive squamous cell carcinoma (SCC) [96]. On clinical examination, OSSN often appears as a fleshy or gelatinous lesion, sometimes with leukoplakic or papillary features, and can be demarcated by vital dye staining with Rose Bengal, toluidine blue, or lissamine green [97].

Slit lamp imaging has been used in multiple AI-based OSSN detection models. Rehman et al. [98] trained CNNs on 634 slit-lamp images, including 163 images of OSSN, 202 images of other ocular surface disorders, and 269 images of normal controls. Ground-truth labels were based on histopathologic confirmation when available and otherwise on the consensus of oculoplastic specialists. During internal validation, the models achieved an average accuracy of 88.8%, with a sensitivity of 74%, a specificity of 96.25%, and an average AUC of 0.92. In a separate study, Ramezani et al. [99] trained a CNN on 162 slit-lamp images (85 images of OSSN vs. 77 images of pterygium). In this case, clinical diagnosis served as the ground truth without histopathologic confirmation. The model achieved an AUC of 0.98 and 94% accuracy, sensitivity, and specificity on internal validation. While the metrics are impressive, the absence of histopathological confirmation limits confidence in the interpretation of these results.

Several classification studies have included OSSN among other ocular surface tumors. Gu et al. [34] trained a model on 5325 slit-lamp images and prospectively validated it on 510 patients. Per-class AUCs were more than 0.91, and overall performance was reported as comparable to that of 10 ophthalmologists. Ground-truth labels were derived from consensus grading by trained ophthalmologists and benchmarked against senior corneal specialists. Ueno et al. [100] used a large multicenter dataset to train a CNN across nine diagnoses. For ocular surface tumors, including OSSN, reported performance was high with an AUC of 0.997. However, the reference standard was not clearly described, and histopathologic confirmation was not reported. In a subsequent reader-assist study, AI support increased clinician accuracy from 79.2% to 88.8%, with 100% correct classification of ocular surface tumors. Ground-truth labels in these studies relied solely on clinical impression, without histopathologic or expert-consensus verification, and OSSN was grouped with diverse entities, including dermoid, lymphoma, pterygium, and cyst. This grouping limits direct clinical comparability and interpretation of disease-specific performance [39,100].

Beyond slit-lamp imaging, advanced modalities such as AS-OCT have been used to differentiate OSSN from benign lesions. High-resolution OCT (HR-OCT) reveals classic OSSN features, such as a hyperreflective, thickened epithelium with an abrupt transition from normal tissue. Quantitative cutoffs based on epithelial thickness have shown excellent diagnostic performance. For example, an epithelial thickness threshold of over 120 μm differentiated OSSN from pterygium with 100% sensitivity and specificity [58,60].

**Figure 3:**
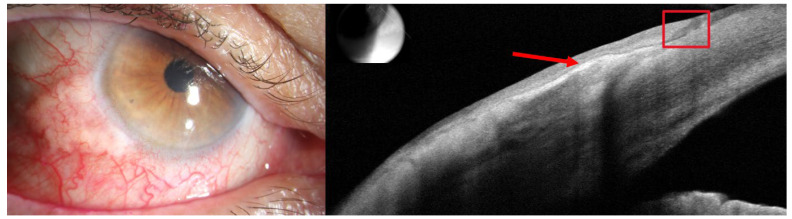
Slit-lamp photograph and corresponding anterior segment optical coherence tomography (AS-OCT) of a subtle ocular surface squamous neoplasia (OSSN) in the left eye of an 80-year-old Hispanic male. The AS-OCT image shows a minimally thickened, hyperreflective epithelial layer (red arrow), with an angled transition to normal epithelium (red box). The lesion was classified as non-OSSN by clinical assessment, whereas the deep learning model identified features consistent with OSSN. This diagnosis was subsequently confirmed by biopsy to be OSSN.

Using the OSSN features, Greenfield et al. [69] developed a deep learning model that differentiates OSSN from pterygium and pinguecula using AS-OCT images. The model was trained on 105,859 unlabeled images from 5746 eyes of 4057 patients and then fine-tuned on 2022 labeled images. Testing was performed against 566 biopsy-proven cases. The model achieved an accuracy of 90.3% with a sensitivity of 86.4%, a specificity of 93.2%, and an AUC of 0.945. These values exceeded those of expert graders, whose accuracy was 86.2%, sensitivity 69.8%, and AUC 0.688. A representative example of this OCT-based AI approach is shown in Figure 3. These results may suggest that OCT-based AI can aid clinicians in challenging differential diagnoses.

IVCM has also been explored as a diagnostic tool for OSSN. Kozma et al. [101] trained a CNN on 2774 images from 97 patients. The dataset included 745 images of OSSN and 1559 images of normal corneas and other ocular surface pathologies. Accuracy ranged from 98% to 99% for binary classification of OSSN. Cell-level analysis of 7501 segmented cells yielded accuracies ranging from 88% to 92%. Histopathologic confirmation following excisional biopsy served as the ground truth for OSSN. The authors noted that IVCM images may contain patient-specific fingerprint features, which raises concerns regarding dataset splitting. A summary of AI-based diagnostic approaches for OSSN is provided in Table 4.

Across slit-lamp photography, OCT, and IVCM, AI models have demonstrated high diagnostic accuracy for OSSN, with OCT-based approaches showing promise in pathology-confirmed settings.

## 9. Gaps and Limitations

Although progress has been encouraging, several obstacles still limit the clinical use of AI models for ocular surface disorders. A recurring issue is scale, with many studies relying on relatively small, single-center datasets. Furthermore, many are limited to a single imaging device, such as a specific OCT platform or slit-lamp camera. This narrow technical context may limit the applications of these findings to everyday practice. In addition, the frequent reliance on case–control designs populated by overtly normal and overtly diseased images may present an overly optimistic picture of performance when compared with the ambiguity of real clinical encounters.

One of the fundamental limitations across ocular surface diseases is that labels in many studies are derived from clinical impression, or expert consensus rather than pathology-confirmed diagnoses. This is particularly relevant for disorders such as DED and IK, where clinical appearances may overlap across entities.

Validation concerns further complicate interpretation. Most reports depend on internal data splits or cross-validation, while prospective testing and true multicenter evaluation remain uncommon. How these models behave when image quality varies, acquisition protocols shift, or patient demographics change is often left unexplored. Methodological reporting is also often selective. Accuracy and AUC are usually foregrounded, whereas calibration, subgroup behavior, or decision-analytic relevance receive far less attention.

Perhaps most notably, demonstrations of clinical impact are rare. Algorithms are typically evaluated in retrospective classification settings, without assessing whether they alter workflow efficiency, shorten diagnostic timelines, influence treatment decisions, or affect patient outcomes.

## 10. Future Directions

Larger, prospective, multicenter datasets appear necessary if models are meant to function outside the narrow settings in which many are currently trained. Patients differ. Devices differ. Even the manner in which images are acquired varies from clinic to clinic. Datasets that fail to reflect this heterogeneity may perform well in testing yet struggle once deployed. Standardized diagnostic definitions could help mitigate some of this variability, particularly when pathology-confirmed ground truth is available for neoplastic disease.

Questions of methodology also remain unresolved. Many investigations report familiar performance metrics, yet fewer address how those numbers were generated or whether protocols were defined in advance. Translation into practice will likely require models that more accurately reflect how clinicians work in practice.

In the future, clinicians should understand that AI systems function as diagnostic guidance or decision-support tools. This may better align with real-world workflows and regulatory expectations. Integrating slit-lamp photography with OCT, IVCM, and biochemical markers may more closely approximate real-world diagnostic reasoning than single-modality approaches. Even then, performance in isolation does not guarantee clinical value. Prospective studies that place AI systems into routine workflows remain scarce, yet they are needed to determine whether human–AI interaction improves efficiency, reduces interobserver variability, or alters management decisions in a meaningful way.

## 11. Conclusions

Artificial intelligence has shown promise in enhancing the diagnostic evaluation of ocular surface disorders, including benign and malignant lesions. Using slit-lamp images, OCT, IVCM, and clinical biomarkers, AI models have achieved accuracy comparable to that of specialists, with higher accuracy in some cases. The strength of AI was in tasks such as meibography grading and OSSN detection. These findings highlight the potential of AI to provide reproducible, scalable diagnostic support across these disorders. However, the evidence still lacks generalizability due to small, device-specific datasets; inconsistent labeling standards; and a low rate of external or prospective validation.

Moving forward, this field needs to address the current gaps. In addition, it is important for prospective trials to evaluate how AI can complement clinicians in real-world workflows, thereby improving efficiency and reducing diagnostic variability. Attention to transparency and ongoing model monitoring will further support safe clinical adoption. By taking these steps, AI models can mature from proof-of-concept tools into robust systems that improve access to ocular surface care and elevate the standard of care in ophthalmology.

## Figures and Tables

**Table 1:** Summary of artificial intelligence-based diagnostic approaches for infectious keratitis.

Reference Number	Study (Year)	AI Task	Imaging Modality	Dataset	Key Performance	Summary
[34]	Gu et al. (2020)	IK detection	Slit-lamp photography	5325 images	AUC 0.939	Effective slit lamp-based infectious keratitis detection, with a potential risk of misclassification with noninfectious corneal disease.
[35]	Tiwari et al. (2022)	Active ulcer vs. scar	Slit-lamp photography	1012 images	AUC 0.973	Accurately differentiates active ulcers from scars using external photographs, but limited to bacterial/fungal IK.
[36]	Redd et al. (2022)	Etiology classification	Slit-lamp photography	980 ulcers	AUC 0.83	Enables etiologic classification of infectious keratitis, with lower performance observed for bacterial pathogens.
[37]	Soleimani et al. (2023)	Triage + subtyping	Slit-lamp photography	980 ulcers	AUC 0.86	Supports triage and subtype classification using task specific slit lamp models rather than a unified diagnostic framework.
[38]	Essalat et al. (2023)	Pathogen detection	IVCM	4001 images	≥90% metrics	Provides high accuracy pathogen detection using in vivo confocal microscopy, limited by modality availability.

**Table 2:** Artificial intelligence-based diagnostic approaches for corneal ectatic disorders.

Reference Number	Study (Year)	AI Task	Dataset	Key Performance	Summary
[45]	Ambrosio Jr. et al. (2017)	Detection of clinical and subclinical ectasia (TBI)	480 normal eyes, 204 keratoconic eyes, 72 very asymmetric ectatic eyes with no surgery, and 94 healthy fellow eyes in patients with very asymmetric ectatic eyes	AUC 0.996	Integrates structural and biomechanical parameters; strong performance for early disease.
[46]	Lopes et al. (2018)	Post-LASIK ectasia risk detection (PRFI)	2980 stable post-LASIK eyes, 45 post-LASIK ectatic eyes, and 182 keratoconic eyes	AUC 0.992	Particularly effective for post-refractive ectasia; sensitivity profile differs from TBI.
[47]	Ambrosio Jr. et al. (2023)	Detection of clinical and subclinical ectasia (TBI v2)	>3800 eyes across expanded ectasia spectrum	AUC 0.999 (clinical); 0.945 (subclinical)	Improves performance over original TBI; broader disease representation.
[48]	Huo et al. (2025)	Ethnicity-specific ectasia detection	492 normal eyes, 247 bilateral keratoconic eyes, 146 very asymmetric ectatic eyes with normal topography, and 127 contralateral ectatic eyes from the asymmetric ectasia group.	AUC >0.90 (ethnicity-dependent)	Highlights population-dependent performance; limited cross-ethnic generalizability.
[49]	Herber et al. (2021)	KC staging	116 normal eyes, 318 keratoconic eyes	AUC ranging from 0.88 to 0.97 among disease severity	Single-center study; staging performance varies by disease severity; no external validation.

**Table 4:** Artificial intelligence-based diagnostic approaches for ocular surface squamous neoplasia.

Reference Number	Study (Year)	AI Task	Imaging Modality	Dataset	Key Performance	Summary
[98]	Rehman et al. (2025)	OSSN detection vs. other ocular surface disease (OSD).	Slit-lamp photography	634 images (OSSN, other OSD, normal)	AUC 0.92; accuracy 88.8%	Demonstrates accurate OSSN detection from slit-lamp images, with partial histopathologic confirmation limiting diagnostic ground truth.
[99]	Ramezani et al. (2025)	OSSN vs. pterygium	Slit-lamp photography	162 images	AUC 0.98	Shows high accuracy distinguishing OSSN from pterygium using slit-lamp photography without histopathologic confirmation.
[34]	Gu et al. (2020)	Ocular surface tumor classification	Slit-lamp photography	5325 images; prospective validation	Per-class AUC >0.91	Enables ocular surface tumor classification from slit-lamp images, with OSSN analyzed as part of a broader tumor category.
[100]	Ueno et al. (2024)	Ocular surface tumor detection	Slit-lamp photography	Multicenter dataset	AUC 0.997	Achieves excellent tumor detection performance across centers, though reference standard definition remains unclear.
[69]	Greenfield et al. (2025)	OSSN vs. benign lesions	AS-OCT	105,859 unlabeled + 2022 labeled scans; 566 biopsy-proven cases	AUC 0.945	Accurately differentiates OSSN from benign lesions using AS-OCT with biopsy-proven histopathologic ground truth.
[101]	Kozma et al. (2025)	OSSN detection	IVCM	2774 images from 97 patients	Accuracy 98–99%	Provides highly accurate OSSN detection using in vivo confocal microscopy, with potential risk of patient-level feature leakage.

## Data Availability

No new data were created or analyzed in this study. Data sharing is not applicable to this article.

## References

[1] Oellers P., Karp C.L., Sheth A., Kao A.A., Abdelaziz A., Matthews J.L., Dubovy S.R., Galor A. (2013). Prevalence, Treatment, and Outcomes of Coexistent Ocular Surface Squamous Neoplasia and Pterygium. Ophthalmology.

[2] Sangwan V.S., Tseng S.C. (2001). New perspectives in ocular surface disorders. An integrated approach for diagnosis and management. Indian. J. Ophthalmol..

[3] Shields C.L., Markowitz J.S., Belinsky I., Schwartzstein H., George N.S., Lally S.E., Mashayekhi A., Shields J.A. (2011). Conjunctival melanoma: Outcomes based on tumor origin in 382 consecutive cases. Ophthalmology.

[4] Stapleton F., Alves M., Bunya V.Y., Jalbert I., Lekhanont K., Malet F., Na K.S., Schaumberg D., Uchino M., Vehof J. (2017). TFOS DEWS II Epidemiology Report. Ocul. Surf..

[5] Ung L., Bispo P.J.M., Shanbhag S.S., Gilmore M.S., Chodosh J. (2019). The persistent dilemma of microbial keratitis: Global burden, diagnosis, and antimicrobial resistance. Surv. Ophthalmol..

[6] Ting D.S.J., Ho C.S., Deshmukh R., Said D.G., Dua H.S. (2021). Infectious keratitis: An update on epidemiology, causative microorganisms, risk factors, and antimicrobial resistance. Eye.

[7] Cicinelli M.V., Marchese A., Bandello F., Modorati G. (2018). Clinical Management of Ocular Surface Squamous Neoplasia: A Review of the Current Evidence. Ophthalmol. Ther..

[8] Cohen V.M.L., O’Day R.F. (2020). Management Issues in Conjunctival Tumours: Ocular Surface Squamous Neoplasia. Ophthalmol. Ther..

[9] Abràmoff M.D., Lou Y., Erginay A., Clarida W., Amelon R., Folk J.C., Niemeijer M. (2016). Improved Automated Detection of Diabetic Retinopathy on a Publicly Available Dataset Through Integration of Deep Learning. Investig. Ophthalmol. Vis. Sci..

[10] Gulshan V., Peng L., Coram M., Stumpe M.C., Wu D., Narayanaswamy A., Venugopalan S., Widner K., Madams T., Cuadros J. (2016). Development and Validation of a Deep Learning Algorithm for Detection of Diabetic Retinopathy in Retinal Fundus Photographs. JAMA.

[11] Craig J.P., Nichols K.K., Akpek E.K., Caffery B., Dua H.S., Joo C.-K., Liu Z., Nelson J.D., Nichols J.J., Tsubota K. (2017). TFOS DEWS II Definition and Classification Report. Ocul. Surf..

[12] Farrand K.F., Fridman M., Stillman I., Schaumberg D.A. (2017). Prevalence of Diagnosed Dry Eye Disease in the United States Among Adults Aged 18 Years and Older. Am. J. Ophthalmol..

[13] Wolffsohn J.S., Benítez-Del-Castillo J.M., Loya-Garcia D., Inomata T., Iyer G., Liang L., Pult H., Sabater A.L., Starr C.E., Vehof J. (2025). TFOS DEWS III: Diagnostic Methodology. Am. J. Ophthalmol..

[14] Yang H.K., Che S.A., Hyon J.Y., Han S.B. (2022). Integration of Artificial Intelligence into the Approach for Diagnosis and Monitoring of Dry Eye Disease. Diagnostics.

[15] Stegmann H., Werkmeister R.M., Pfister M., Garhöfer G., Schmetterer L., Dos Santos V.A. (2020). Deep learning segmentation for optical coherence tomography measurements of the lower tear meniscus. Biomed. Opt. Express.

[16] Nejat F., Eghtedari S., Alimoradi F. (2024). Next-Generation Tear Meniscus Height Detecting and Measuring Smartphone-Based Deep Learning Algorithm Leads in Dry Eye Management. Ophthalmol. Sci..

[17] Wang K., Xu K., Chen X., He C., Zhang J., Li F., Xiao C., Zhang Y., Wang Y., Yang W. (2025). Artificial intelligence-assisted tear meniscus height measurement: A multicenter study. Quant. Imaging Med. Surg..

[18] Nichols K.K., Nichols J.J., Zadnik K. (2000). Frequency of dry eye diagnostic test procedures used in various modes of ophthalmic practice. Cornea.

[19] Shimizu E., Ishikawa T., Tanji M., Agata N., Nakayama S., Nakahara Y., Yokoiwa R., Sato S., Hanyuda A., Ogawa Y. (2023). Artificial intelligence to estimate the tear film breakup time and diagnose dry eye disease. Sci. Rep..

[20] Abdelmotaal H., Hazarbasanov R., Taneri S., Al-Timemy A., Lavric A., Takahashi H., Yousefi S. (2023). Detecting dry eye from ocular surface videos based on deep learning. Ocul. Surf..

[21] Kikukawa Y., Tanaka S., Kosugi T., Pflugfelder S.C. (2023). Non-invasive and objective tear film breakup detection on interference color images using convolutional neural networks. PLoS ONE.

[22] Stapleton F., Argüeso P., Asbell P., Azar D., Bosworth C., Chen W., Ciolino J.B., Craig J.P., Gallar J., Galor A. (2025). TFOS DEWS III: Digest. Am. J. Ophthalmol..

[23] Wang J., Yeh T.N., Chakraborty R., Yu S.X., Lin M.C. (2019). A Deep Learning Approach for Meibomian Gland Atrophy Evaluation in Meibography Images. Transl. Vis. Sci. Technol..

[24] Fineide F., Storås A.M., Chen X., Magnø M.S., Yazidi A., Riegler M.A., Utheim T.P. (2022). Predicting an unstable tear film through artificial intelligence. Sci. Rep..

[25] Zheng Q., Wang L., Wen H., Ren Y., Huang S., Bai F., Li N., Craig J.P., Tong L., Chen W. (2022). Impact of Incomplete Blinking Analyzed Using a Deep Learning Model with the Keratograph 5M in Dry Eye Disease. Transl. Vis. Sci. Technol..

[26] Yokoi N., Georgiev G.A. (2018). Tear Film-Oriented Diagnosis and Tear Film-Oriented Therapy for Dry Eye Based on Tear Film Dynamics. Investig. Ophthalmol. Vis. Sci..

[27] Yokoi N., Kusada N., Kato H., Furusawa Y., Sotozono C., Georgiev G.A. (2023). Dry Eye Subtype Classification Using Videokeratography and Deep Learning. Diagnostics.

[28] Villani E., Baudouin C., Efron N., Hamrah P., Kojima T., Patel S.V., Pflugfelder S.C., Zhivov A., Dogru M. (2014). In vivo confocal microscopy of the ocular surface: From bench to bedside. Curr. Eye Res..

[29] Zhou S., Robertson D.M. (2018). Wide-Field In Vivo Confocal Microscopy of Meibomian Gland Acini and Rete Ridges in the Eyelid Margin. Investig. Ophthalmol. Vis. Sci..

[30] Maruoka S., Tabuchi H., Nagasato D., Masumoto H., Chikama T., Kawai A., Oishi N., Maruyama T., Kato Y., Hayashi T. (2020). Deep Neural Network-Based Method for Detecting Obstructive Meibomian Gland Dysfunction with in Vivo Laser Confocal Microscopy. Cornea.

[31] Amouei Sheshkal S., Gundersen M., Alexander Riegler M., Aass Utheim Ø., Gunnar Gundersen K., Rootwelt H., Prestø Elgstøen K.B., Lewi Hammer H. (2024). Classifying Dry Eye Disease Patients from Healthy Controls Using Machine Learning and Metabolomics Data. Diagnostics.

[32] Ting D.S.J., Foo V.H., Yang L.W.Y., Sia J.T., Ang M., Lin H., Chodosh J., Mehta J.S., Ting D.S.W. (2021). Artificial intelligence for anterior segment diseases: Emerging applications in ophthalmology. Br. J. Ophthalmol..

[33] Ting D.S.J., Gopal B.P., Deshmukh R., Seitzman G.D., Said D.G., Dua H.S. (2022). Diagnostic armamentarium of infectious keratitis: A comprehensive review. Ocul. Surf..

[34] Gu H., Guo Y., Gu L., Wei A., Xie S., Ye Z., Xu J., Zhou X., Lu Y., Liu X. (2020). Deep learning for identifying corneal diseases from ocular surface slit-lamp photographs. Sci. Rep..

[35] Tiwari M., Piech C., Baitemirova M., Prajna N.V., Srinivasan M., Lalitha P., Villegas N., Balachandar N., Chua J.T., Redd T. (2022). Differentiation of Active Corneal Infections from Healed Scars Using Deep Learning. Ophthalmology.

[36] Redd T.K., Prajna N.V., Srinivasan M., Lalitha P., Krishnan T., Rajaraman R., Venugopal A., Acharya N., Seitzman G.D., Lietman T.M. (2022). Image-Based Differentiation of Bacterial and Fungal Keratitis Using Deep Convolutional Neural Networks. Ophthalmol. Sci..

[37] Soleimani M., Esmaili K., Rahdar A., Aminizadeh M., Cheraqpour K., Tabatabaei S.A., Mirshahi R., Bibak-Bejandi Z., Mohammadi S.F., Koganti R. (2023). From the diagnosis of infectious keratitis to discriminating fungal subtypes; a deep learning-based study. Sci. Rep..

[38] Essalat M., Abolhosseini M., Le T.H., Moshtaghion S.M., Kanavi M.R. (2023). Interpretable deep learning for diagnosis of fungal and acanthamoeba keratitis using in vivo confocal microscopy images. Sci. Rep..

[39] Maehara H., Ueno Y., Yamaguchi T., Kitaguchi Y., Miyazaki D., Nejima R., Inomata T., Kato N., Chikama T.-i., Ominato J. (2025). Artificial intelligence support improves diagnosis accuracy in anterior segment eye diseases. Sci. Rep..

[40] Taki Y., Ueno Y., Oda M., Kitaguchi Y., Ibrahim O.M.A., Aketa N., Yamaguchi T. (2024). Analysis of the performance of the CorneAI for iOS in the classification of corneal diseases and cataracts based on journal photographs. Sci. Rep..

[41] Prajna N.V., Assaf J., Acharya N.R., Rose-Nussbaumer J., Lietman T.M., Campbell J.P., Keenan J.D., Song X., Redd T.K. (2025). Multimodal Deep Learning for Differentiating Bacterial and Fungal Keratitis Using Prospective Representative Data. Ophthalmol. Sci..

[42] Duncan J.K., Esquenazi I., Weikert M.P. (2017). New Diagnostics in Corneal Ectatic Disease. Int. Ophthalmol. Clin..

[43] Belin M.W., Jang H.S., Borgstrom M. (2022). Keratoconus: Diagnosis and Staging. Cornea.

[44] Lin S.R., Ladas J.G., Bahadur G.G., Al-Hashimi S., Pineda R. (2019). A Review of Machine Learning Techniques for Keratoconus Detection and Refractive Surgery Screening. Semin. Ophthalmol..

[45] Ambrósio R., Lopes B.T., Faria-Correia F., Salomão M.Q., Bühren J., Roberts C.J., Elsheikh A., Vinciguerra R., Vinciguerra P. (2017). Integration of Scheimpflug-Based Corneal Tomography and Biomechanical Assessments for Enhancing Ectasia Detection. J. Refract. Surg..

[46] Lopes B.T., Ramos I.C., Salomão M.Q., Guerra F.P., Schallhorn S.C., Schallhorn J.M., Vinciguerra R., Vinciguerra P., Price F.W., Price M.O. (2018). Enhanced Tomographic Assessment to Detect Corneal Ectasia Based on Artificial Intelligence. Am. J. Ophthalmol..

[47] Ambrósio R., Machado A.P., Leão E., Lyra J.M.G., Salomão M.Q., Esporcatte L.G.P., da Fonseca Filho J.B.R., Ferreira-Meneses E., Sena N.B., Haddad J.S. (2023). Optimized Artificial Intelligence for Enhanced Ectasia Detection Using Scheimpflug-Based Corneal Tomography and Biomechanical Data. Am. J. Ophthalmol..

[48] Huo Y., Xie R., Li J., Hou J., Zou H., Wang Y. (2025). Ethnicity optimized indices enhance the diagnostic efficiency of early Keratoconus: A multicenter validation study. Contact Lens Anterior Eye.

[49] Herber R., Pillunat L.E., Raiskup F. (2021). Development of a classification system based on corneal biomechanical properties using artificial intelligence predicting keratoconus severity. Eye Vis..

[50] Tiong E.W.W., Liu S.-H., Ting D.S.J. (2024). Cochrane corner: Artificial intelligence for keratoconus. Eye.

[51] Jaros P.A., DeLuise V.P. (1988). Pingueculae and pterygia. Surv. Ophthalmol..

[52] Chui J., Di Girolamo N., Wakefield D., Coroneo M.T. (2008). The pathogenesis of pterygium: Current concepts and their therapeutic implications. Ocul. Surf..

[53] Perkins E.S. (1985). The association between pinguecula, sunlight and cataract. Ophthalmic Res..

[54] Yam J.C., Kwok A.K. (2014). Ultraviolet light and ocular diseases. Int. Ophthalmol..

[55] Mackenzie F.D., Hirst L.W., Battistutta D., Green A. (1992). Risk analysis in the development of pterygia. Ophthalmology.

[56] Lucas R.M. (2011). An epidemiological perspective of ultraviolet exposure—Public health concerns. Eye Contact Lens.

[57] Dushku N., Hatcher S.L., Albert D.M., Reid T.W. (1999). p53 expression and relation to human papillomavirus infection in pingueculae, pterygia, and limbal tumors. Arch. Ophthalmol..

[58] Kieval J.Z., Karp C.L., Abou Shousha M., Galor A., Hoffman R.A., Dubovy S.R., Wang J. (2012). Ultra-high resolution optical coherence tomography for differentiation of ocular surface squamous neoplasia and pterygia. Ophthalmology.

[59] Shousha M.A., Karp C.L., Canto A.P., Hodson K., Oellers P., Kao A.A., Bielory B., Matthews J., Dubovy S.R., Perez V.L. (2013). Diagnosis of ocular surface lesions using ultra-high-resolution optical coherence tomography. Ophthalmology.

[60] Nanji A.A., Sayyad F.E., Galor A., Dubovy S., Karp C.L. (2015). High-Resolution Optical Coherence Tomography as an Adjunctive Tool in the Diagnosis of Corneal and Conjunctival Pathology. Ocul. Surf..

[61] Herskowitz W.R., De Arrigunaga S., Greenfield J.A., Cohen N.K., Galor A., Karp C.L. (2025). Can high-resolution optical coherence tomography provide an optical biopsy for ocular surface lesions?. Can. J. Ophthalmol..

[62] Agarwal A., Kaliki S., Murthy S.I. (2023). Corneal squamous neoplasia: Masquerades and management outcomes at a rural eyecare centre. BMJ Case Rep..

[63] Gokharu S., Arya D., Chauhan D., Das S. (2025). Ocular surface squamous neoplasia masquerades: Clinical profile and outcome. Indian J. Ophthalmol..

[64] Huang J.J., Locatelli E.V.T., Huang J.J., De Arrigunaga S., Rao P., Dubovy S., Karp C.L., Galor A. (2024). It Is All About the Angle: A Clinical and Optical Coherence Tomography Comparison of Corneal Ocular Surface Squamous Neoplasia and Corneal Pannus. Cornea.

[65] Mejía L.F., Zapata M., Gil J.C. (2021). An Unexpected Incidence of Ocular Surface Neoplasia on Pterygium Surgery. A Retrospective Clinical and Histopathological Report. Cornea.

[66] Theotoka D., Wall S., Galor A., Sripawadkul W., Khzam R.A., Tang V., Sander D.L., Karp C.L. (2022). The use of high resolution optical coherence tomography (HR-OCT) in the diagnosis of ocular surface masqueraders. Ocul. Surf..

[67] Hung K.H., Lin C., Roan J., Kuo C.F., Hsiao C.H., Tan H.Y., Chen H.C., Ma D.H., Yeh L.K., Lee O.K. (2022). Application of a Deep Learning System in Pterygium Grading and Further Prediction of Recurrence with Slit Lamp Photographs. Diagnostics.

[68] Wan C., Mao Y., Xi W., Zhang Z., Wang J., Yang W. (2023). DBPF-net: Dual-branch structural feature extraction reinforcement network for ocular surface disease image classification. Front. Med..

[69] Greenfield J.A., Scherer R., Alba D., De Arrigunaga S., Alvarez O., Palioura S., Nanji A., Bayyat G.A., da Costa D.R., Herskowitz W. (2025). Detection of Ocular Surface Squamous Neoplasia Using Artificial Intelligence with Anterior Segment Optical Coherence Tomography. Am. J. Ophthalmol..

[70] Kim J.H., Kim Y.J., Lee Y.J., Hyon J.Y., Han S.B., Kim K.G. (2023). Automated histopathological evaluation of pterygium using artificial intelligence. Br. J. Ophthalmol..

[71] Tiong E.W.W., Soon C.Y.S., Ong Z.Z., Liu S.H., Qureshi R., Rauz S., Ting D.S.J. (2025). Deep learning for diagnosing and grading pterygium: A systematic review and meta-analysis. Comput. Biol. Med..

[72] Mesquita R.G., Figueiredo E.M.N. An algorithm for measuring pterygium’s progress in already diagnosed eyes. Proceedings of the 2012 IEEE International Conference on Acoustics, Speech and Signal Processing (ICASSP).

[73] Wan Zaki W.M.D., Mat Daud M., Abdani S.R., Hussain A., Mutalib H.A. (2018). Automated pterygium detection method of anterior segment photographed images. Comput. Methods Programs Biomed..

[74] Zhang K., Liu X., Liu F., He L., Zhang L., Yang Y., Li W., Wang S., Liu L., Liu Z. (2018). An Interpretable and Expandable Deep Learning Diagnostic System for Multiple Ocular Diseases: Qualitative Study. J. Med. Internet Res..

[75] Saad A., Zamani N., Zaki W., Baseri Huddin A., Hussain A. (2019). Automated Pterygium Detection in Anterior Segment Photographed Images using Deep Convolutional Neural Network. Int. J. Adv. Trends Comput. Sci. Eng..

[76] Zulkifley M.A., Abdani S.R., Zulkifley N.H. (2019). Pterygium-Net: A deep learning approach to pterygium detection and localization. Multimed. Tools Appl..

[77] Zamani N.S.M., Zaki W.M.D.W., Huddin A.B., Hussain A., Mutalib H.A., Ali A. (2020). Automated Pterygium Detection Using Deep Neural Network. IEEE Access.

[78] Abdani S.R., Zulkifley M.A., Zulkifley N.H. (2021). Group and Shuffle Convolutional Neural Networks with Pyramid Pooling Module for Automated Pterygium Segmentation. Diagnostics.

[79] Xu W., Jin L., Zhu P.Z., He K., Yang W.H., Wu M.N. (2021). Implementation and Application of an Intelligent Pterygium Diagnosis System Based on Deep Learning. Front. Psychol..

[80] Zheng B., Liu Y., He K., Wu M., Jin L., Jiang Q., Zhu S., Hao X., Wang C., Yang W. (2021). Research on an Intelligent Lightweight-Assisted Pterygium Diagnosis Model Based on Anterior Segment Images. Dis. Markers.

[81] Fang X., Deshmukh M., Chee M.L., Soh Z.D., Teo Z.L., Thakur S., Goh J.H.L., Liu Y.C., Husain R., Mehta J.S. (2022). Deep learning algorithms for automatic detection of pterygium using anterior segment photographs from slit-lamp and hand-held cameras. Br. J. Ophthalmol..

[82] Gan F., Chen W.Y., Liu H., Zhong Y.L. (2022). Application of artificial intelligence models for detecting the pterygium that requires surgical treatment based on anterior segment images. Front. Neurosci..

[83] Zhu S., Fang X., Qian Y., He K., Wu M., Zheng B., Song J. (2022). Pterygium Screening and Lesion Area Segmentation Based on Deep Learning. J. Healthc. Eng..

[84] Zamani N.S.M., Zaki W.M.D.W., Huddin A.B., Mutalib H.A., Hussain A. (2023). Pterygium Classification Using Deep Patch Region-based Anterior Segment Photographed Images. J. Kejuruter..

[85] Kumar H.S.V., Jayaram M.A. Machine Learning Techniques for Severity Assessment of Pterygium. Proceedings of the 2023 International Conference on Integrated Intelligence and Communication Systems (ICIICS).

[86] Liu Y., Xu C., Wang S., Chen Y., Lin X., Guo S., Liu Z., Wang Y., Zhang H., Guo Y. (2024). Accurate detection and grading of pterygium through smartphone by a fusion training model. Br. J. Ophthalmol..

[87] Luo X., Lin X., Ouyang W., Zheng S., Chen J., Liu Z. (2024). Bi-DenseNet: Automatic recognition of ocular surface disease using smartphone imaging. Biomed. Signal Process. Control..

[88] Ticlavilca-Inche E.J., Moreno-Lozano M.I., Castañeda P.S., Wong-Durand S., Oñate-Andino A. (2024). Mobile Application Based on Convolutional Neural Networks for Pterygium Detection in Anterior Segment Eye Images at Ophthalmological Medical Centers. Int. J. Online Biomed. Eng..

[89] Wu M.N., He K., Yu Y.B., Zheng B., Zhu S.J., Hong X.Q., Xi W.Q., Zhang Z. (2024). Intelligent diagnostic model for pterygium by combining attention mechanism and MobileNetV2. Int. J. Ophthalmol..

[90] Moreno-Lozano M.I., Ticlavilca-Inche E.J., Castaneda P., Wong-Durand S., Mauricio D., Onate-Andino A. (2024). A Performance Evaluation of Convolutional Neural Network Architectures for Pterygium Detection in Anterior Segment Eye Images. Diagnostics.

[91] Ji Q., Liu W., Ma Q., Qu L., Zhang L., He H. (2025). A semantic segmentation-based automatic pterygium assessment and grading system. Front. Med..

[92] Li Z., Wang Z., Xiu L., Zhang P., Wang W., Wang Y., Chen G., Yang W., Chen W. (2025). Large language model-based multimodal system for detecting and grading ocular surface diseases from smartphone images. Front. Cell Dev. Biol..

[93] Koç İ., Kıratlı H. (2020). Current Management of Conjunctival Melanoma Part 1: Clinical Features, Diagnosis and Histopathology. Turk. J. Ophthalmol..

[94] Yoo T.K., Choi J.Y., Kim H.K., Ryu I.H., Kim J.K. (2021). Adopting low-shot deep learning for the detection of conjunctival melanoma using ocular surface images. Comput. Methods Programs Biomed..

[95] Mosquera-Zamudio A., Launet L., Tabatabaei Z., Parra-Medina R., Colomer A., Oliver Moll J., Monteagudo C., Janssen E., Naranjo V. (2022). Deep Learning for Skin Melanocytic Tumors in Whole-Slide Images: A Systematic Review. Cancers.

[96] Lee G.A., Hirst L.W. (1995). Ocular surface squamous neoplasia. Surv. Ophthalmol..

[97] Sayed-Ahmed I.O., Palioura S., Galor A., Karp C.L. (2017). Diagnosis and Medical Management of Ocular Surface Squamous Neoplasia. Expert. Rev. Ophthalmol..

[98] Rehman O., Gujar R., Kumawat R., Pandey R., Gupta C., Tiwari S., Sangwan V., Das S. (2025). Deep Learning-Based Detection of Ocular Surface Squamous Neoplasia from Ocular Surface Images. Ocul. Oncol. Pathol..

[99] Ramezani F., Azimi H., Delfanian B., Amanollahi M., Saeidian J., Masoumi A., Farrokhpour H., Khalili Pour E., Khodaparast M. (2025). Classification of ocular surface diseases: Deep learning for distinguishing ocular surface squamous neoplasia from pterygium. Graefe’s Arch. Clin. Exp. Ophthalmol..

[100] Ueno Y., Oda M., Yamaguchi T., Fukuoka H., Nejima R., Kitaguchi Y., Miyake M., Akiyama M., Miyata K., Kashiwagi K. (2024). Deep learning model for extensive smartphone-based diagnosis and triage of cataracts and multiple corneal diseases. Br. J. Ophthalmol..

[101] Kozma K., Janki Z.R., Bilicki V., Csutak A., Szalai E. (2025). Artificial intelligence to enhance the diagnosis of ocular surface squamous neoplasia. Sci. Rep..

